# Dietary Oleocanthal Supplementation Prevents Inflammation and Oxidative Stress in Collagen-Induced Arthritis in Mice

**DOI:** 10.3390/antiox10050650

**Published:** 2021-04-23

**Authors:** Tatiana Montoya, Marina Sánchez-Hidalgo, María Luisa Castejón, María Ángeles Rosillo, Alejandro González-Benjumea, Catalina Alarcón-de-la-Lastra

**Affiliations:** 1 Department of Pharmacology, School of Pharmacy, Universidad de Sevilla, 41012 Seville, Spain; tmontoya@us.es (T.M.); hidalgosanz@us.es (M.S.-H.); mcastejon1@us.es (M.L.C.); rosillo@us.es (M.Á.R.); 2 Department of Organic Chemistry, School of Chemistry, Universidad de Sevilla, 41012 Seville, Spain; a.g.benjumea@irnas.csic.es

**Keywords:** diet, inflammation, oleocanthal, oxidative stress, rheumatoid arthritis, secoiridoid

## Abstract

Oleocanthal (OLE), a characteristic and exclusive secoiridoid of *Oleoaceae* family, is mainly found in extra virgin olive oil (EVOO). Previous studies have reported its antioxidant, anti-inflammatory, antimicrobial, anticancer and neuroprotective effects. Since the pathogenesis of rheumatoid arthritis (RA) involves inflammatory and oxidative components, this study was designed to evaluate the preventive role of dietary OLE-supplemented effects in collagen-induced arthritis (CIA) murine model. Animals were fed with a preventive OLE-enriched dietary during 6 weeks previous to CIA induction and until the end of experiment time. At day 43 after first immunization, mice were sacrificed: blood was recollected and paws were histological and biochemically processed. Dietary OLE prevented bone, joint and cartilage rheumatic affections induced by collagen. Levels of circulatory matrix metalloproteinase (MMP)-3 and pro-inflammatory cytokines (IL-6, IL-1β, TNF-α, IL-17, IFN-γ) were significantly decreased in secoiridoid fed animals. Besides, dietary OLE was able to diminish COX-2, mPGES-1 and iNOS protein expressions and, also, PGE_2_ levels. The mechanisms underlying these protective effects could be related to Nrf-2/HO-1 axis activation and the inhibition of relevant signaling pathways including JAK-STAT, MAPKs and NF-κB, thus controlling the production of inflammatory and oxidative mediators. Overall, our results exhibit preliminary evidences about OLE, as a novel dietary tool for the prevention of autoimmune and inflammatory disorders, such as RA.

## 1. Introduction

Rheumatoid arthritis (RA) is a chronic systemic inflammatory disease characterized by severe synovitis leading to progressive joints destruction accompanied by systemic inflammation. RA is often considered an autoimmune condition expressing certain characteristic autoantibodies such as rheumatoid factors and antibodies to citrullinated proteins [1].

The etiology of RA involves genetics and environmental factors association. Nevertheless, geography, socioeconomic status, diet/nutrients, alcohol, smoking, and host-microbiome also contribute to the risk of developing RA [2,3].

Although the pathogenesis of RA is multifactorial, various inflammatory pathways lead to an altered immune function. Particularly, there are massive immune cells infiltration in the synovial membrane, including CD4^+^ T, B and natural killer cells, in addition to macrophages, dendritic cells, neutrophils and mast cells. All of them induce aberrant cytokines production such as tumor necrosis factor alpha (TNF)-α, interferon (IFN)-γ and interleukins (IL) (IL-6, IL-8, IL-12/23, IL-17 or IL-18), which perpetuate and ease joint inflammation, rising matrix metalloproteases (MMPs), and consequently activating osteoclasts at joint site. As a result, pannus invades subchondral tissue, destroying cartilage and generating ankyloses [4]. Furthermore, RA includes extra-articular manifestations, such as rheumatoid protuberances, pulmonary involvement or vasculitis and systemic comorbidities [5].

The possible processes that promote the expression of these disease inductors involve several signal transduction pathways, as the signal transducer and activator of transcription (JAK-STAT) activated in response to pro-inflammatory cytokines by steering the abnormal activation, automaticity, and prolonged survival of synovial cells; mitogen-activated protein kinases (MAPKs), which comprise extracellular signal-regulated kinases (ERK), c-Jun N-terminal kinases (JNK) and p38; and, nuclear transcription factor-kappa B (NF-κB) [6]. In particular, NF-κB plays an important role in MMPs production and, also, modulates a wide range of genes that contribute to inflammation, primarily IL-1β, TNF-α, IL-6, chemokines and the inducible enzymes cyclooxygenase-2 (COX-2) and inducible nitric oxide synthase (iNOS) [7,8]. Likewise, a reduction of antioxidant defense system, mostly marked by an increment of oxidation products and DNA damage, has been described in RA [9]. In this context, nuclear factor E2-related factor 2 (Nrf-2), a key transcription factor orchestrator that maintains the cellular defense against oxidative stress, acts inducing expression of detoxifying enzymes and antioxidant proteins as heme oxygenase-1 (HO-1), which is decreased in RA patient’s acute joints [10,11].

Accumulating research evidence suggests that individual dietary factors and patterns might be implicated in the risk of RA development. A balanced diet and its nutrients exhibit antioxidant, anti-inflammatory and immunomodulatory properties. The Mediterranean diet (MD) rich in plant-based foods such as whole grains, legumes, fruit, vegetables and extra-virgin olive oil (EVOO), might have the potential to reduce RA. One of the main MD characteristic habits is the daily consumption of 25–50 g/day of EVOO [12].

The antioxidant and anti-inflammatory properties of the biophenolic fraction from *Olea europaea* L leaves and fruits have been suggested for their potential applications in a large number of inflammatory and reactive oxygen species (ROS)-mediated diseases. In fact, the olive tree is rich in phenolic compounds predominantly the secoiridoids: oleuropein, dimethyl-oleuropein, ligstroside, and their hydrolysis derivatives such as oleuropein-aglycone, oleocanthal (OLE), oleacein, elenolate, oleoside-11-methyl ester, elenoic acid, hydroxytyrosol and tyrosol [13].

Since OLE had been published in the literature for the first time in 1993 [14], it has attracted increasing attention of experts. Initially, OLE was known to be responsible for particular pungency and irritative sensation in the throat and oropharyngeal area when swallowing olive oil. The OLE-linked pungent sensation seems to be mediated via the activation of transient receptor potential cation channel subtype A1 (TRPA1), which is known to mediate neurogenic and chronic inflammation [15]. Nevertheless, OLE is getting more scientific attention due to its interesting biological activities, most antioxidant, anti-inflammatory, antimicrobial, anticancer and neuroprotective effects [13,16]*,* even though it makes up only 10% of the olive’s polyphenols (0.2 mg/kg up to 498 mg/kg) influenced by several different factors such as geographical location, olive fruit maturity, agricultural techniques, oil extraction and storage conditions, or even cooking procedures [16]. It is noteworthy that diet consumption of 25–50 mL EVOO provides around 10 mg of OLE daily, corresponding to near 10% of ordinary ibuprofen dose [15,16].

Up-to-day, few clinical trials and in vivo experimental models have been performed, so data about bioavailability and metabolism of OLE are still reduced. It has been suggested that OLE absorption goes on in the small intestine, thanks to its propitious partition coefficient (log *p* = 1.02) [17]. Moreover, OLE may remain stable at gastric acid conditions for 4 h [18]. OLE is mainly metabolized by Phase I (hydrogenation, hydroxylation, and hydration), but also some hydrogenated metabolites may pass to Phase II of metabolism as glucoronidated forms. In fact, this secoridioid and its metabolites were found in human urine after the ingestion of 50 mL of VOO [19,20,21] and in plasma samples after acute ingestion of 30 mL of EVOO [22]. However, definitive elucidation of the pharmacokinetic profile of OLE should be a future focus of research.

Essentially, OLE exerts several protective functions in neurodegeneration through its ability to modulate oxidative stress and apoptosis in neuronal cells [23]*,* suggesting it as a potential adjunct to drugs against Alzheimer and Parkinson diseases [17]. Besides, a large number of studies supports its anticancer potential tested on different in vitro and in vivo cancer assays [17,24,25]. Complementary, other bioactive properties in individual health have been attributed to this secoiridoid as cardio-protection and antimicrobial actions [13,18,26].

Indeed, OLE has shown potent anti-inflammatory effects inhibiting the activities of COX-1 and COX-2, and also 5-lipoxygenase (5-LOX), the rate-determining enzymes for the synthesis of prostaglandins (PGs) and pro-inflammatory leukotrienes, respectively. Surprisingly, OLE exerted better anti-inflammatory activity than the non-steroidal anti-inflammatory drug, (NSAID), ibuprofen, at the same concentration. These results promoted later studies with OLE, which were useful to assign its health-benefiting properties, suggesting OLE as a possible key supplement to several inflammatory diseases and improving the side-effects of classic pharmacological therapy [18,27,28,29]. Recently, our research group has demonstrated the potential anti-inflammatory and antioxidant modulation of OLE in lipopolysaccharide (LPS)-stimulated murine peritoneal macrophages which represents a well-established model for validate anti-inflammatory drugs [30].

Although the studies about the antioxidant activity of OLE are very limited, it has been demonstrated that OLE could inhibit nicotinamide adenine dinucleotide phosphate oxidase (NOX) in isolated human monocytes, and diminish intracellular ROS levels in SH-SY5Y cells [13].

In terms of rheumatic diseases, OLE has shown to ameliorate osteoarthritis and RA in vitro. However, to date, there are not data about the possible beneficial effects of OLE for an in vivo model of RA. Thus, this study was designed to evaluate an OLE-enriched diet preventive effects on collagen-induced arthritis (CIA) murine model. For this purpose, we analyzed macroscopic and histological damage, production of inflammatory mediators and, explored possible signaling pathways involved.

## 2. Materials and Methods

### 2.1. Chemicals

OLE was isolated from EVOO Cornicabra cultivar (1000 g). Oil was mixed with acetonitrile (400 mL), the mixture was shaken vigorously in a separating funnel and the two phases were allowed to separate. The process was repeated with extra acetonitrile (2 × 400 mL) to ensure complete extraction of phenols.

The combined polar phases were concentrated to an oily residue using a rotary evaporator at residue pressure. The residue was washed with cyclohexane (2 × 10 mL) to eliminate residual triglycerides, and purified by column chromatography using silica gel 60 (40–63 mm) (Merck^®^, Darmstadt, Germany), as the stationery phase and a gradient EtOAc/cyclohexane (0:1 → 1:2) as the eluent.

The purity of the extracted OLE was based on the ^1^H and ^13^C NMR spectra and HPLC analyses. NMR spectra were registered in CDCl3 in a Bruker Avance-300 spectrometer; ^1^H-RMN: (300 MHz, CDCl_3_): δ (ppm) 9.62 (1H, s, H-3); 9.22 (1H, d, *J*_1,8_ = 2.0 Hz, H-1); 7.0 (2H, m, H-4′, H-8′); 6.76 (2H, m, H-5′, H-7′); 6.63 (1H, q, *J*_8,10_ = 7.1 Hz, H-8); 4.22 (1H, dt, *J*_1a’,1b’_ = 10.7 Hz, *J*_1a’,2′_ = 7.0 Hz, H-1a’); 4.18 (1H, dt, *J*_1b’,1a’_ = 10.7 Hz, *J*_1b’,2′_ = 7.0 Hz, H-1b’); 3.6 (1H, m, H-5); 2.97 (1H, ddd, *J*_4a,4b_ = 18.4 Hz, *J*_4a,5_ = 8.7 Hz, *J*_4a,3_ = 1.3 Hz, H-4a); 2.81 (2H, t, *J*_2′,1′_ = 7.0 Hz, H-2′); 2.76 (1H, ddd, *J*_4b,4a_ = 18.4 Hz, *J*_4b,5_ = 6.62 Hz, *J*_4b,3_ = 0.85 Hz, H-4b); 2.69 (1H, dd, *J*_6a,6b_ = 15.8 Hz, *J*_6a,5_ = 8.2 Hz, H-6a); 2.60 (1H, dd, *J*_6b,6a_ = 15.8 Hz, *J*_6b,5_ = 7.0 Hz, H-6b); 2.06 (3H, d, *J*_10,8_ = 7.1 Hz, H-10). ^13^C-RMN: (75.5 MHz, CDCl_3_): δ(ppm) 200.7 (C-3); 195.4 (C-1); 172.2 (C-7); 154.7 (C-6′); 154.6 (C-8); 143.4 (C-9); 130.3 (C-3′); 130.2 (C-4′, C-8′); 115.5 (C-5′, C-7′); 65.3 (C-1′); 46.3 (C-4); 37.0 (C-6); 34.3 (C-2′); 27.4 (C-5); 15.4 (C-10). HPLC analysis was performed in a Waters 600 system, equipped with a Waters 2996 PDA detector and a C18 column (Nova-Pak, 4 μm, 3.9 × 150 mm column). Mobile phase consisted of a gradient of 2% acetic acid in water (A) and acetonitrile (B). The gradient, at a flow rate of 1.0 mL/min, was as follows: 2 min from 100% to 95% of A; 8 min from 95% to 75% of A; 10 min from 75% to 60% of A; 10 min from 60% to 50% of A. The analyses showed that the isolated OLE had purity higher than 95% (data are shown in Supplementary Materials).

### 2.2. Animals and Diet

Thirty-four three-weeks-old male DBA-1/j mice (Janvier^®^, Le Genest St Isle, France) were housed under standard conditions (24–25 °C, humidity 70–75%, lighting regimen 12L/12D) in our Animal Laboratory Center. All mice were acclimated for 4 weeks prior to the initiation of experiments, feeding with standard diet (SD) and water ad libitum. At 4-weeks-aged animals were divided into three experimental groups: (1) Naïve group received a SD (*n* = 10), (2) CIA group received a SD (SD-CIA) (*n* = 12) and, (3) CIA-OLE group received a SD supplemented with OLE 0.025% (dietary enrichment percentage selection based on our previous reports [31,32]) (*n* = 12). All diets were elaborated according to the basis of the American Institute of Nutrition (AIN) standard reference diet, mixing OLE or no, and stored them at –80 °C. Experimental groups were fed with fresh diet daily during six weeks, previously to first immunization and until the day of sacrifice. Animal care and procedures agreed with protocols approved by Animal Ethics Committee of the Universidad de Sevilla (ethical approval number 23/07/2018/119), and recommendations of European Union regarding animal experimentation (Directive of the European Counsel 2012/707/EU).

### 2.3. Collagen Type II Induction of Arthritis Disease

The first immunization of disease was injected at the basis of the tail when DBA-1/j mice were ten-weeks-old (Day O). Bovine type II collagen (CII) was diluted in acetic acid (2 mg/mL) (MD Bioproducts^®^, Zurich, Switzerland) and was emulsified with an equal volume of complete Freund’s adjuvant (2 mg/mL *Mycobacterium tuberculosis,* strain H37Ra; Difco^®^, Detroit, MI, USA). On day 21, a second intraperitoneal immunization was injected with 100 mg of CII dissolved in phosphate buffered saline (PBS).

### 2.4. Clinical Assessment of Joints

The development of arthritis was considered and evaluates daily when mice exhibited redness and/or swelling affections in digits, joints or entire paws. To evaluate the arthritic index for each paw of mice, a macroscopic score system was used: 0, no signs; 1, mild inflammation of joint; 1.5, marked inflammation of joint and digits; 2, severe inflammation of paw. Scoring was performed by two observers-blind.

### 2.5. Histological Analysis

On day 43, mice were sacrificed and knees joints were removed and fixed in 4% formalin. Paws were decalcified in 10% EDTA for 30 days and, then, were dehydrated and embedded in paraffin. Samples sectioned (7 mm) were stained with hematoxylin and eosin (H&E) or tartrate-resistant acid phosphate (TRAP) using naphtol-AS-MX phosphate (Sigma^®^, St. Louis, MO, USA) and fat red violet LB salt (Sigma^®^, St. Louis, MO, USA), to perform histological analysis.

### 2.6. Immunohistochemical Detection of COX-2 Expression

For immunohistochemistry assay of COX-2, the procedure was according to Rosillo et al. (2014) [31]. Joints-sectioned (7 mm) were prepared from paraffin-embedded tissues. To avoid unspecific staining, deparaffined sections was inhibited with hydrogen peroxide and incubated in normal horse serum (Vectastain Kit^®^; Vector Laboratories, Burlingame, CA, USA). After 20 min, slides were incubated with rabbit anti-COX-2 (1:600) (Cell Signaling Technology^®^, Danvers, MA, USA) overnight at 4 °C. Then, they were incubated for 30 min with antirabbit IgG antibody (Vectastain Kit^®^; Vector Laboratories, Burlingame, CA, USA), following by 30 min streptavidin-peroxidase complex incubation. Finally, slides were treated with the peroxidase substrate 3,3-diaminobenzidine and water-washed, counterstaining with hematoxylin. To negative control, slides followed the same procedure without primary antibody incubation.

### 2.7. Enzyme-Linked Immunosorbent Assay

MMP-3 was measured in serum using an ELISA kit with 10 pg/mL of sensitivity (R&D System^®^, Abingdon, UK). To determinate levels of pro-inflammatory cytokines, hind paws were homogenized in liquid N_2_ with A buffer, as previously described Rosillo et al. (2014) [31]. Supernatants obtained were used to quantify production of IL-6, IFN-γ (Diaclone, Besacon Cedez, France), TNF-α, IL-1β (R&D System^®^, Abingdon, UK) and IL-17 (Peprotech^®^, London, UK).

### 2.8. Western Blotting

Hind paws were homogenized in liquid N_2_ with a phosphate buffer according to Rosillo et al., (2014), in order to isolate cytoplasmic and nuclear proteins [31]. Protein concentration was determined with Bradford’s colorimetric method [33]. Nuclear fraction was used to measure p50 and p65 expression and, cytoplasmic fraction was used for the rest of determinations. A total of 50 μg of proteins were separated on 10% sodium dodecyl sulphatepolyacrylamide gel electrophoresis, transferred to nitrocellulose membrane and, incubated overnight at 4 °C with specific primary antibodies: COX-2, iNOS, pJNK, JNK, pp38, p38, pERK 1/2, ERK 1/2, Nrf-2, pSTAT-3, IκB-α, p50, p65 (Cell Signaling Technology^®^, Danvers, MA, USA), HO-1 (Henzo^®^, Madrid, Spain), mPGES-1 (Abcam^®^, Cambridge, MA, USA). Membranes were incubated for 2 h at room temperature with horseradish peroxidase-labeled secondary antibody antirabbit (Cell Signaling Technology^®^, Danvers, MA, USA) or antimouse (Dako^®^, Atlanta, GA, USA). Blots were analyzed with β-actin antibody (Abcam^®^, Cambridge, MA, USA) to prove equal loading. The immunosignals were captured with Amersham Image 600 from GE Healthcare (Buckinghamshire, UK) and signals were analyzed and quantified using Image Processing and Analysis in Java (ImageJ, Softonic).

### 2.9. Data Analysis

All values are expressed as arithmetic means ± standard error (SEM) in figures and text. Results were evaluated using Graph Pad Prism version 5.01 software (San Diego, CA, USA), analyzing the statistical significance by one-way analysis of variance (ANOVA), followed by Tukey’s multiple comparisons test. *p-*Values < 0.05 were considered statistically significant. Figures from densitometry experiments are representatives of different experiments performing on a different day.

## 3. Results

### 3.1. Effects of OLE Supplemented Diet on Collagen-Induced Arthritis Model

The similarity of CIA and RA reside in the loss of tolerance and self-antigens production against collagen, one of the main self-antigens also observed in human RA. Immunization of mice with an emulsion of CII on complete Freund’s adjuvant exhibits an erosive polyarthritis with an autoimmune response against cartilage mediated by both auto-reactive B and T cells. Consequently, these pathogenic antibodies detect their endogenous antigen in the joint triggering a local inflammatory response, synovial inflammatory cell infiltration, hyperplasia, cartilage destruction and bone erosion [34].

**Figure 1:**
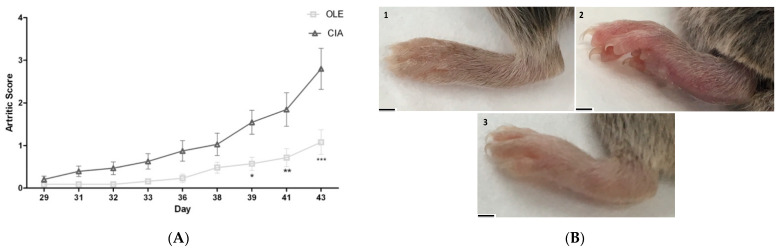
Evolution of arthritis severity in collagen-induced arthritis (CIA) mice from day 29 to 43 after second immunization. (**A**) Changes in paw swelling and thickness on arthritic mice. Sings was scored every two days up to the end of experiment. CIA control group, collagen-induced arthritis mice fed with standard diet (SD); OLE, arthritic mice fed with 0.025% *w/w* oleocanthal-supplemented diet. Values are presented as mean ± S.E.M. (*n* = 10). * *p* < 0.05; ** *p* < 0.01; *** *p* < 0.001 vs. CIA group. (**B**) Image of hind paws at the end of experiment (Day 43): Naïve group, non-arthritic mouse fed with SD; CIA control group with SD; and, OLE supplemented diet arthritic group. Scale bar = 1 cm (1–3).

The model requires at least 6–8 weeks for the appreciation of clinical sings consistently. Indeed, the sings of arthritis were evaluated since day 29. We observed a progressive development of clinical symptoms and swelling from day 33 in CIA control mice fed with a normal standard diet (SD-CIA) (Figure 1A).

On the contrary, animals that were fed with OLE showed a decrease in arthritis severity, reducing footpad thickness and inflammation of forelegs and hind legs in comparison with SD-CIA group. Representative images of hind paws from different experimental animal groups supported these results. In effect, OLE dietary not only retarded the development, but also exhibited an effective therapeutic activity on disease onset (Figure 1B).

**Figure 2:**
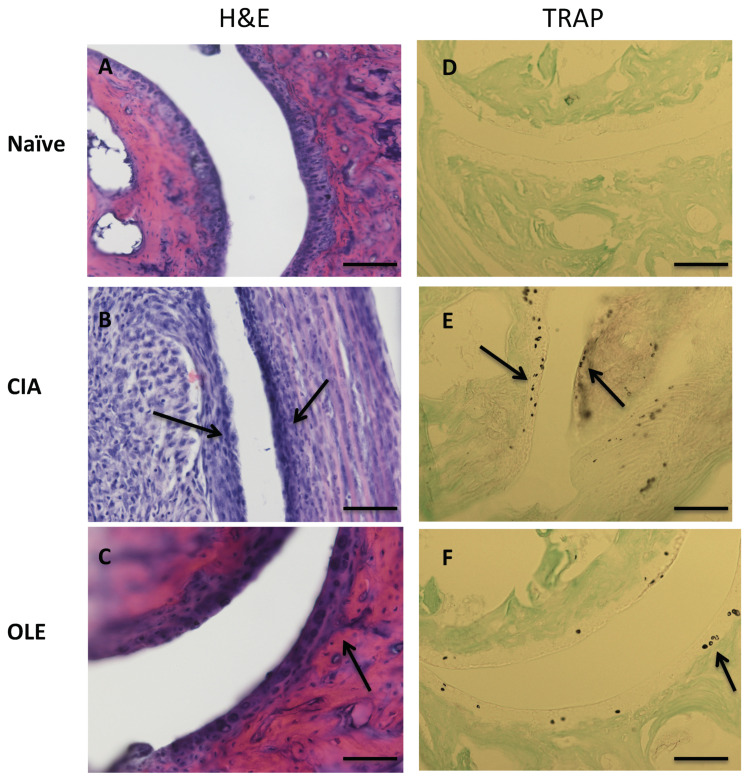
Histological representative images of frontal joint section at Day 43: effects of dietary OLE (0.025% *w/w*) in synovial tissue, cartilage damage and bone erosion. Paw section from mice (*n* = 10) was stained with H&E (**A**–**C**) to show inflammatory activity and TRAP (**D**–**F**) staining for bone erosion. (**A**,**D**) Naïve group, non-arthritic control animals fed with SD; (**B**,**E**) CIA, induced arthritic animals fed with SD; (**C**,**F**) OLE, induced arthritic animals fed with OLE enriched diet. Joint cartilage thinning due to arthritic damage (asterisk) and inflammatory infiltrates (arrow) were observed in SD-CIA mice (**B**), as well as increased osteoclast cells (arrow) (**E**). Original magnification ×100 (**A**–**C**) and ×200 (**D**–**F**). The pictures are representative of at least six independent experiments with similar results.

As shown in Figure 2, H&E staining revealed infiltration of inflammatory cells into articular tissues, exudation into the synovial tissue, synovial hyperplasia and cartilage and bone damage in SD-CIA group, compared to Naïve group. However, the histological features of joints from mice fed with OLE resembled Naïve control (Figure 2A–C). TRAP, is considered to be just a histochemical marker of osteoclasts and is expressed by osteoclasts, macrophages, dendritic cells and a number of other cell types [35]. Consequently, TRAP staining showed larger quantities of osteoclasts and bone erosion in joint sections from CIA mice whereas joint sections from mice fed with OLE diet presented a significant reduction of TRAP-positive osteoclasts (Figure 2D–F).

### 3.2. Effects of Dietary OLE on MMP-3 and Joint Inflammatory Biomarkers Levels

**Figure 3:**
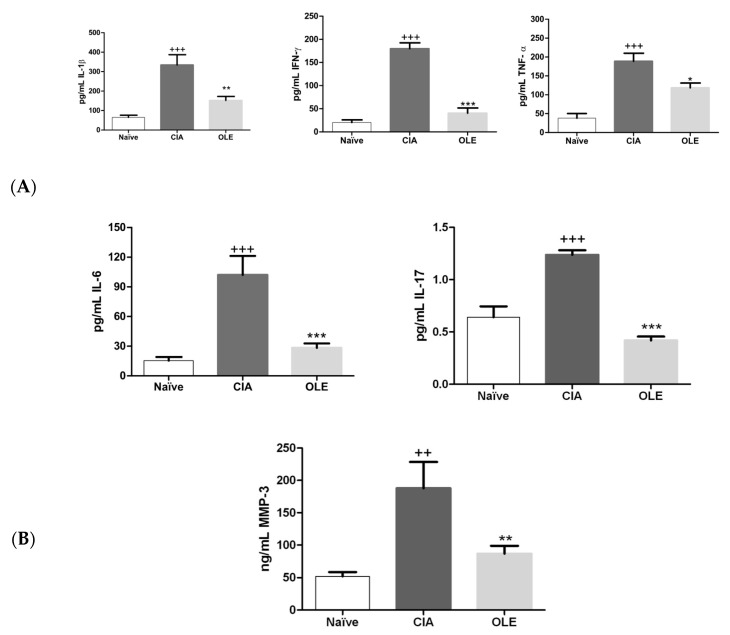
Effects of dietary oleocanthal on MMP-3 and joint inflammatory biomarkers levels. (**A**) OLE diet down-regulated the levels of pro-inflammatory cytokines: IL-1β, IFN-γ, IL-6, IL-17 and TNF-α in tissue homogenates; (**B**) MMP-3 levels was decreased in serum of animals fed with OLE diet. Naïve group, non-arthritic control animals fed with SD; CIA, control group induced arthritic animals fed with SD; OLE induced arthritic animals fed with OLE enriched diet (0.025% *w/w*). Values are represented as mean ± S.E.M. (*n* = 8), ^++^ *p* < 0.01; ^+++^ *p* < 0.001 vs. Naïve group; * *p* < 0.05; ** *p* < 0.01; *** *p* < 0.001 vs. CIA group.

It has been reported that pro-inflammatory cytokines as TNF-α, IL-1β, IFN-γ, IL-6, or IL-17 play a critical role on the pathogenesis of RA [36]. Besides, pro-inflammatory cytokines, including TNF-α, IL-1β and IL-6, may activate MMP genes through the binding of several different transcription factors [37]. In order to address whether OLE-supplemented diet could regulate these joint inflammatory biomarkers, we measured cytokine levels in paw homogenates and MMP-3 in serum. As shown in Figure 3A, IL-1β, IFN-γ, TNF-α, IL-6 and IL-17 levels were markedly increased in paw homogenates from SD-CIA animals in comparison with sham group suggesting its relationship with the synovial tissue inflammation (*p* < 0.001 vs. Naïve). Nevertheless, our data indicated a significant reduction of pro-inflammatory cytokines production in paw homogenates from animals fed with OLE-diet (IL-1β: *p* < 0.01; IFN-γ: *p* < 0.001; TNF-α: *p* < 0.05; IL-6: *p* < 0.001; IL-17: *p* < 0.001 vs. CIA) (Figure 3A).

In addition, circulating levels of MMP-3 were raised in SD-CIA animals, in parallel to the disease severity (*p* < 0.01 vs. Naïve) (Figure 3B). On the contrary, a significant reduction of MMP-3 levels was observed in OLE-enriched diet fed animals *(p* < 0.01 vs. CIA).

### 3.3. Effects of Dietary OLE on COX-2 Protein, mPGES-1 Expression and PGE_2_ Production

**Figure 4:**
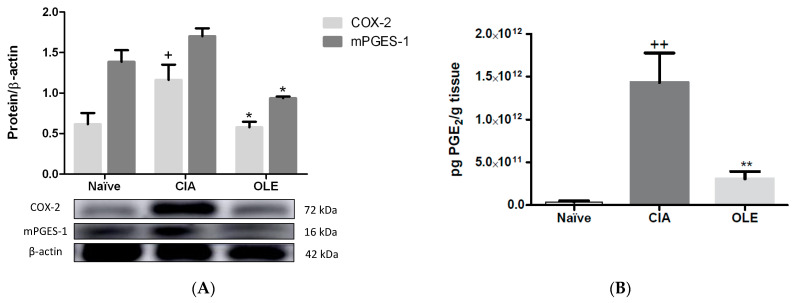
Modulation of COX-2/mPGES-1 axis and PGE_2_ synthesis in dietary OLE supplemented group in hind paws. (**A**) COX-2 and mPGES-1 protein expression were analyzed by Western blotting and quantified by densitometrical analysis. (**B**) Quantification of PGE_2_ levels using ELISA kit. Western blot images are representative of separate experiments with similar results. Naïve group, non-arthritic control animals fed with SD; CIA, control group induced arthritic animals fed with SD; OLE induced arthritic animals fed with OLE enriched diet (0.025% *w/w*). Values represented mean ± S.E.M. (*n* = 8), ^+^ *p* < 0.05; ^++^ *p* < 0.01 vs. Naïve control group; * *p* < 0.05; ** *p* < 0.01 vs. CIA group.

**Figure 5:**
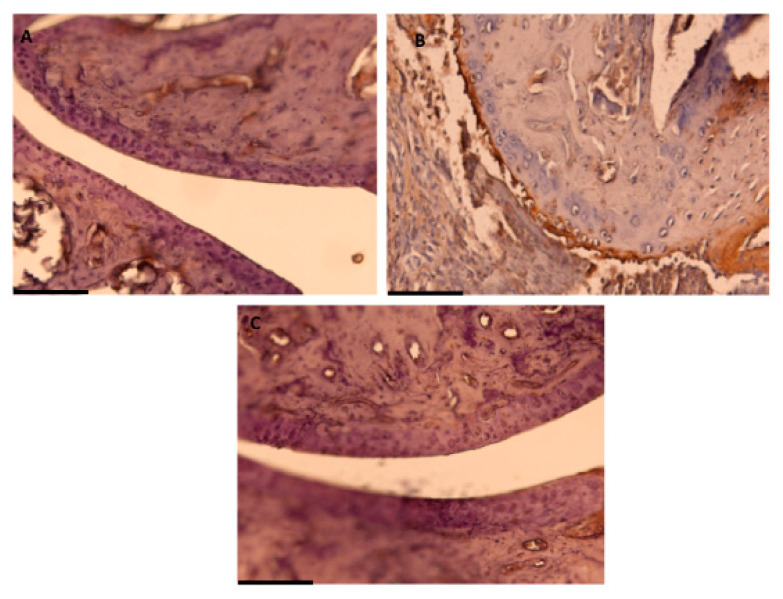
Effect of dietary OLE on COX-2 expression through immunochemistry analysis. Brown color indicates specific immunopositive staining for COX-2. (**A**) COX-2 expression in Naïve group fed with SD with minor or no positive staining. (**B**) Joint section of CIA group fed with SD showing marked expression of COX-2. (**C**) OLE-enriched diet group presenting marked less or no COX-2 immunopositive staining. COX-2 protein production was evaluated in joint sections of different experimental groups. Original magnification ×200 (**A**–**C**). (**A**–**C**) Pictures are representative of at least six independent experiments with similar results.

COX-2 and mPGES-1 expressions and levels of PGE_2_ were determined by Western blot and ELISA, respectively, in paw homogenates. Additionally, COX-2 expression was studied by immunohistochemistry in joints sections (Figure 4 and Figure 5).

Arthritic control animal group showed an overexpression of both pro-inflammatory enzymes (COX-2: *p* < 0.01; mPGES-1: *p* < 0.001 vs. Naïve) in parallel with an increase of PGE_2_ levels (*p* < 0.01 vs. Naïve). On the contrary, dietary OLE was able to diminish the protein expression of both proteins (COX-2: *p* < 0.01; mPGES-1: *p <* 0.001 vs. CIA) and PGE_2_ levels (*p* < 0.01 vs. CIA) in CIA-induced mice (Figure 4).

Similar results were found after the immunohistochemical analysis. We could observe overexpression of COX-2 positive cells in SD-CIA control group, whereas OLE experimental diet reduced remarkably the immunoreactivity for this pro-inflammatory enzyme (Figure 5).

### 3.4. OLE Experimental Diet Attenuated iNOS Overexpression

**Figure 6:**
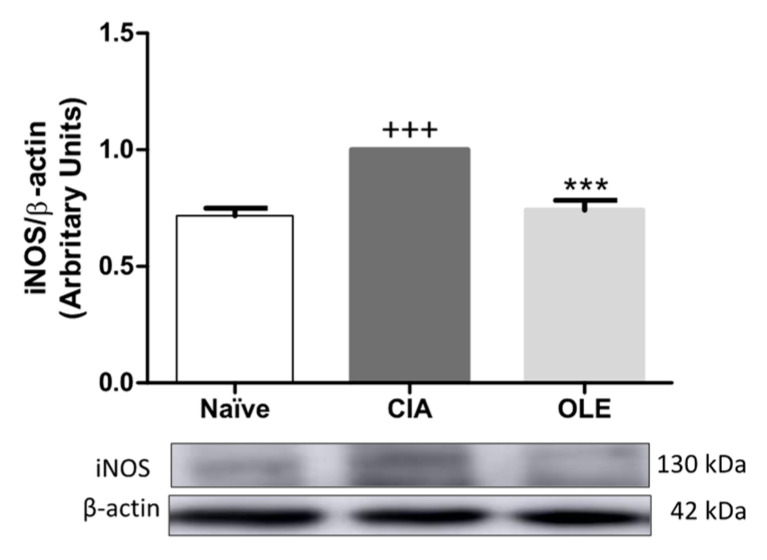
Protein expression of iNOS in hind paws homogenate. Protein expression was quantified by densitometry analysis and normalized with respect to β-actin (house-keeping). Western blot images are representative of separate experiments with similar results. Naïve group, non-arthritic control animals fed with SD; CIA, control group induced arthritic animals fed with SD; OLE induced arthritic animals fed with OLE enriched diet (0.025% *w/w*). Values represented mean ± S.E.M (*n* = 8), ^+++^ *p* < 0.001 vs. Naïve control group; *** *p* < 0.001 vs. CIA group.

iNOS is considered a critical oxidative stress marker involved in nitric oxide (NO) production, a free radical inductor of oxidation [8]. Down-regulation of iNOS expression was observed in hind paw of CIA mice group submitted to treatment with OLE-supplemented diet when compared with CIA mice received SD diet (*p* < 0.001 vs. CIA), as shown in Figure 6.

### 3.5. Effects of Dietary OLE on pSTAT-3 Protein Expression

**Figure 7:**
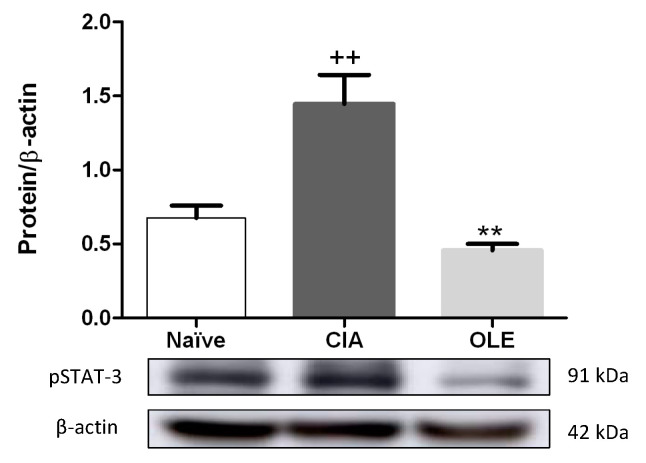
OLE supplemented diet regulated pSTAT-3 in hind paws homogenates from arthritis induced mice. The expression of pSTAT-3 was measured by Western blotting and normalized with respect to β-actin (house-keeping). Western blot images are representative of separate experiments with similar results. Naïve group, non-arthritic control animals fed with SD; CIA, control group induced arthritic animals fed with SD; OLE induced arthritic animals fed with OLE enriched diet (0.025% *w/w*). Values represented mean ± S.E.M (*n* = 8), ^++^ *p* < 0.01 vs. Naïve control group; ** *p* < 0.01 vs. CIA group.

STAT-3 has been described as a critical transcription factor involved in the pathogenesis of RA and is activated by upstream cytokines, such as IL-6 and IL-17, among others [38]. We evaluated pSTAT-3 protein expression by Western blot from hind paw homogenates. Statistical analysis revealed that pSTAT-3 was overexpressed in CIA group compared with Naïve group (*p* < 0.01 vs. Naïve) whereas, nutritional therapy with OLE significantly suppressed STAT-3 phosphorylation in arthritic CIA mice (*p* < 0.01 vs. CIA) (Figure 7).

### 3.6. Dietary OLE Induces Nrf-2/HO-1 Antioxidant Pathway Activation

**Figure 8:**
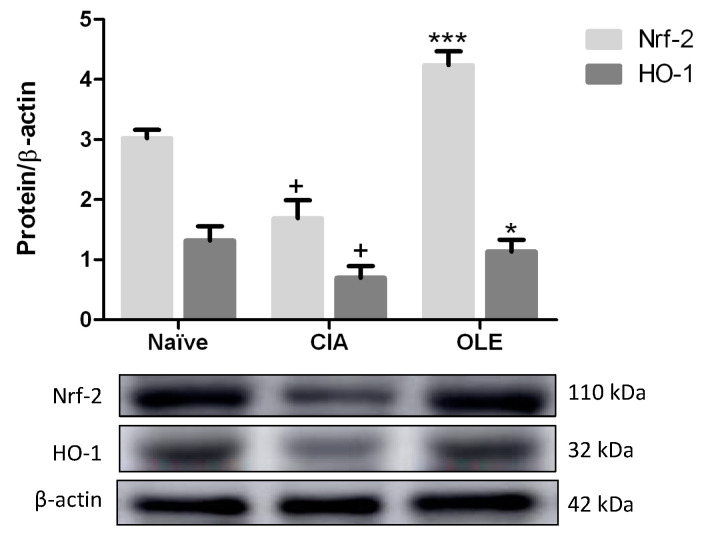
OLE dietary treatment induced up-regulation of Nrf-2/HO-1 protein expression in hind paws homogenates. Densitometry analysis was normalized with respect to β-actin (house-keeping). Western blot images are representative of separate experiments with similar results. Naïve group, non-arthritic control animals fed with SD; CIA, control group induced arthritic animals fed with SD; OLE induced arthritic animals fed with OLE enriched diet (0.025% *w/w*). Values represented mean ± S.E.M. (*n* = 8), ^+^ *p* < 0.05 vs. Naïve control group; * *p* < 0.05; *** *p* < 0.001 vs. CIA group.

The expression of the proteins HO-1 and Nrf-2 were also evaluated in paw homogenates by Western blotting. Balance of Nrf-2 maintains the regulation of oxidative stress, inflammation, immune response, and cartilage and bone metabolism through activation of antioxidant enzymes, as HO-1. Thus, Nrf-2 regulates redox status and plays key roles in cellular defense by enhancing the removal of ROS [10]. As shows Figure 7, CIA mice fed with SD diet presented a reduced expression of both proteins, in comparison with Naïve control group (*p <* 0.05 vs. Naïve). However, a significant overexpression of both proteins, Nrf-2 and HO-1, were observed in paw homogenates from animals fed with OLE diet (*p <* 0.001 and *p <* 0.05 vs. CIA, respectively) (Figure 8).

### 3.7. OLE Diet Modulates MAPKs Signaling Pathway

**Figure 9:**
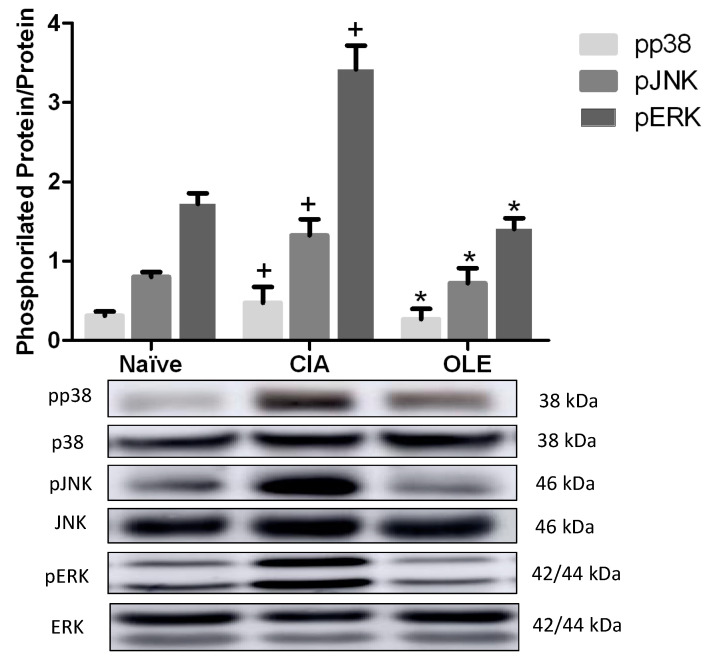
OLE diet modulated MAPKs signaling pathways in hind paws homogenates. Phosphorylated p38, JNK and ERK was analyzed with Western blot and normalized with respect to specific non-phosphorylated total proteins. Western blot images are representative of separate experiments with similar results. Naïve group, non-arthritic control animals fed with SD; CIA, control group induced arthritic animals fed with SD; OLE induced arthritic animals fed with OLE enriched diet (0.025% *w/w*). Values represented mean ± S.E.M. (*n* = 8), ^+^ *p* < 0.05 vs. Naïve non-arthritic group; * *p* < 0.05 vs. CIA arthritic group.

It has been reported an important role of MAPKs in the RA pathogenesis, inducing pro-inflammatory gene expression which initiates inflammatory responses [39]. In the present study, we measured phosphorylation, and therefore, activation of p38, ERK and JNK MAPKs. Our data showed that phosphorylation of JNK, p38 and ERK proteins were increased significantly in cytosolic extracts from SD-CIA mice paw homogenates, compared with Naïve animals (*p <* 0.05 vs. Naïve). Again, OLE diet showed its effectiveness along the experimental period. In fact, the protein expressions of all phosphorylated MAPKs proteins, p-JNK, p-p38 and p-ERK, were significantly improved after dietary OLE treatment (*p* < 0.05 vs. CIA) (Figure 9).

### 3.8. Effects of Dietary OLE on NF-κB Signaling Pathway

We analyzed the preventive effects of OLE supplemented diet on IκB-α degradation and nuclear translocation of the subunits p65 and p50 from paw homogenates. Firstly, we observed that in SD-CIA animals’ group, IκB-α expression was decreased, revealing degradation, and therefore allowing NF-κB translocation into the nucleus (*p* < 0.05 vs. Naïve). Contrary, IκB-α was overexpressed in cytoplasmic extracts from mice fed with OLE diet, so there was not degradation (*p* < 0.05 vs. CIA).

**Figure 10:**
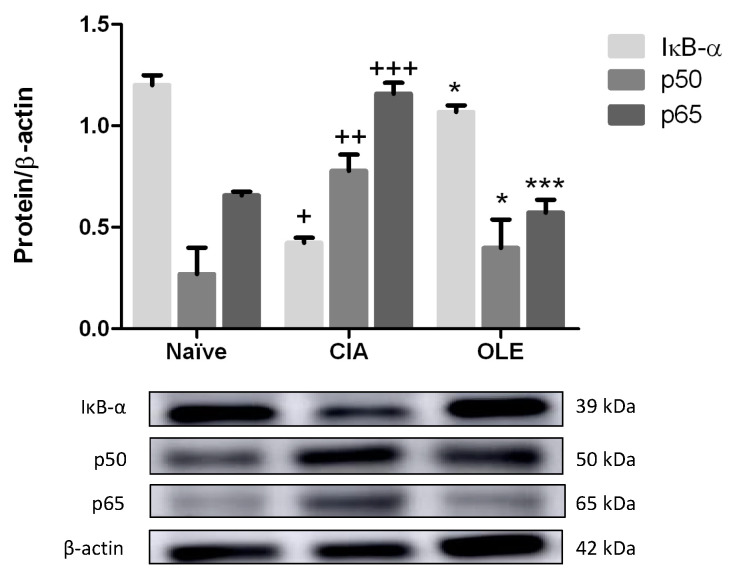
OLE diet-enriched controlled NF-κB signaling pathway preventing IκB-α degradation and p65 and p50 subunits migration to the nucleus. The expression was quantified by densitometry analysis and normalized with respect to β-actin (house-keeping). Western blot images are representative of separate experiments with similar results. Naïve group, non-arthritic control animals fed with SD; CIA, control group induced arthritic animals fed with SD; OLE induced arthritic animals fed with OLE enriched diet (0.025% *w/w*). Values represented mean ± S.E.M. (*n* = 8), ^+^ *p* < 0.05; ^++^ *p* < 0.01; ^+++^ *p* < 0.001 vs. Naïve group; * *p* < 0.05; *** *p* < 0.001 vs. CIA group.

These results were corroborated, after analysis of both p65 and p50 subunits in nucleus extracts from paw homogenates. As shown Figure 10, the nuclear p65 and p50 protein expressions were significantly increased in CIA group (p50: *p* < 0.01; p65: *p* < 0.001 vs. Naïve); however, dietary OLE treatment could prevent the CIA-induced nuclear translocation of both subunits in comparison with arthritic animals fed with SD (p50: *p* < 0.05; p65: *p* < 0.001 vs. CIA), avoiding the NF-κB-mediated transcriptional activation (Figure 10).

## 4. Discussion

Our study has revealed, for the first time, a preventive role of OLE-enriched diet on the arthritic process through a CIA murine model. This is a useful model to study and evaluate important RA pathogenic mechanisms and novel antiarthritic drugs. It was first described in 1977 and is characterized by the production of CII-specific antibodies [40], a feature that has also been described in RA.

In RA or CIA, the normal organization of synovium appears imbalanced and becoming infiltrated with T and B cells, macrophages and neutrophils [41]. This fact triggers a hyperplasic membrane of synoviocytes that invades and destroys bone and cartilage. Conversely, dietary OLE supplementation could control the arthritis score through a lowering infiltration of immune cells, an appreciable reduction of oedema, synovial hyperplasia, cartilage degradation and bone erosion compared with CIA mice fed with a SD.

The development and progression of RA are closely related to production of MMPs, pro-inflammatory cytokines and oxidation products. Th-17 cells produce cytokines such as IL-17, considered a major player in the pathogenesis of CIA. IL-17 promotes inflammation by enhancing the production of crucial pro-inflammatory cytokines, mainly TNF-α, IL-1β, IL-6 and RANLK, among others [42]. At the pannus cartilage junction, these pro-inflammatory mediators can induce the production of additional cytokines, chemokines and MMPs, assisting cartilage degradation [43]. Moreover, TNF-α activates osteoclastogenesis, suppresses the osteoblasts recruitment and inhibits the expression of matrix genes [44]. Additionally, IL-1β is one of best serum markers of RA patients, which is correlated with disease activity and implicated in degradation and destruction of matrix cartilage and articular joints. Likewise, IL-6 can be detected in synovial fluid of RA patients. This cytokine not only acts as a growth factor for T and B cells and, antibody production but also, induces synoviocytes proliferation, osteoclast differentiation and increment of MMPs levels [43].

In terms of MMPs, MMP-3 is a proteinase secreted by synovial fibroblasts and chondrocytes in the synovium and, is responsible for the degradation of proteoglycan, various type of collagen types, fibronectin, aggrecan core protein, among others [32,45]. Our results agree with above studies and showed that high IL-1β, IL-6, IFN-γ, IL-17, TNF-α and MMP-3 levels were associated with disease onset and joint progression in CIA mice. However, arthritic mice fed with OLE-enriched diets showed a significant reduction in serum MMP-3 levels as well as in IL-17, TNF-α, IL1-β, IFN-γ and IL-6 pro-inflammatory cytokines in paw homogenates in comparison with CIA control group, correlating in parallel with the macroscopic and histological findings. Therefore, these data suggest that dietary OLE exerts anti-inflammatory effects through the modulated production of these key RA biomarkers. Similar data were found in our recent study, which reported that OLE controlled the production of inflammatory mediators decreasing IL-1β, TNF-α, INF-γ, IL-6, IL-17 and IL-18 in LPS-stimulated murine peritoneal macrophages [30] and, in previous works reported by Scotece et al., (2012; 2019) using a chondrogenic and macrophage cell line or human primary osteoarthritis (OA) chondrocytes LPS-stimulated [28,29].

RA patients present a significant ROS production [46]. This inflammation-associated action could induce osteoclast differentiation that could act in feedback way rising ROS levels [47]. Moreover, IL-1β, IL-6 or TNF-α promote osteoclasts formation. Although the presence of osteoclasts in bone loss has been scarcely documented in RA, it has been widely accepted as responsible for bone erosion in RA patients [48]. Actually, TRAP staining is considered one of the best histochemical markers of osteoclasts and Suzuki et al. described TRAP staining–positive multinucleated cells in CIA as a validated method [49]. Our data showed that joint sections from mice fed with OLE diet presented a marked reduction of TRAP-positive osteoclasts, suggesting a decrease of osteoclast activity in OLE-supplemented diet-fed mice.

To further evaluate the antioxidant role involved in OLE effects, iNOS expression was studied. iNOS is an inducible enzyme implicates in NO production of RA patients, inducing oxidative damage of arthritic joint [50]. Several works have reported how iNOS expression was induced in murine models of CIA, relating to NF-κB signaling pathway [8,32,51]. In our study, OLE-enriched diet showed comparable results to iNOS modulation in LPS-induced NO and ROS production, and iNOS overexpression in murine peritoneal macrophages [30]. Consequently, these data suggest that dietary OLE treatment could attenuate incidence of CIA through down-regulation of NF-kB and iNOS expression, regulating oxidative damage of cartilage and improving clinical signs.

COX-2 and mPGES-1, enzymes responsible for the overproduction of PGE_2_ implicated in inflammation and pain hypersensitivity associated with RA, are up-regulated contributing to the disease progression through EP_4_ receptor activation [45,52]. COX-2 enzyme expression is induced by cytokines such as TNF-α, IL-1β and IL-6. Our data are in concordance with other authors who reported increased levels of COX-2 in CIA models [32,45,53]. In the present study, we have demonstrated that dietary OLE treatment reduced COX-2 expression in knee joints from DBA/1j mice CIA model. These results are in accordance with those reported by Montoya et al. (2019) in LPS-induced murine peritoneal macrophages [30]*,* in human monocytes stimulated with LPS [54] and in LPS-activated human primary OA chondrocytes [29]. Complementary, we have shown that dietary OLE supplementation reduced mPGES-1 expression, which was accompanied by a significant decrease in PGE_2_ levels possibly due to decreased expression of COX-2 in the joint. Therefore, regulation of these pro-inflammatory biomarkers could represent a potential molecular target susceptible to OLE modulation that has not been previously established in vivo.

Signal transduction pathways strongly implicated in inflammation and oxidative stress include MAPKs, JAK-STAT and NF-κB. NF-κB, a dimeric transcription factor, activates multiple pro-inflammatory genes expression in the microenvironment of arthritic joints, such as Th-1 and Th-17, COX-2, iNOS, in addition to MAPKs [7,26]. In resting cells, NF-κB, p65 and p50 heterodimers composition is present in cytoplasm blocked by inhibitor protein IκB-α. However, pro-inflammatory stimulus undergoes phosphorylation and, subsequently, ubiquitination, then allowing NF-κB p65 and p50 heterodimers migrate to the nucleus and bind to specific promoter sequences [7]. Consequently, suppression of the NF-κB pathway could be a novel strategy for delaying the progress of RA. Our data agree with Rosillo et al. (2016), who reported that, in control CIA mice, IκB-α was significantly degraded and p65 and p50 subunits were translocated to the nucleus [55]. Nevertheless, dietary OLE treatment prevented nuclear p65- and p50-NF-κB translocations by blocking IκB-α degradation. These facts resulted in an ameliorated pro-inflammatory markers production and, then, reducing the joint inflammatory injury. Similar data were found recently by Carpi et al. (2019) who investigated the impact of OLE on NF-κB the activation and the expression of molecules associated with inflammatory and dysmetabolic responses in Simpson–Golabi–Behmel syndrome adipocytes TNF-α-stimulated. OLE attenuated the activation of NF-κB pathway and prevented NF-κB activity by directly interacting with the p65 subunit [56]. Complementary, LPS-induced inflammation in human primary OA chondrocytes also exhibited a significant regulation of IκB and NF-κB-p65 subunit after OLE treatment [29].

MAPK family members, including p38 kinases, ERK and JNK, play critical roles in many important cell processes, including inflammation. They have been detected in tissue from RA patients, suggesting an implication in pathogenesis of this disease [39]. Phosphorylated forms of MAPKs can induce transcriptional and post-transcriptional activation of different cytokines involved in RA. Moreover, ERK promotes pannus formation; JNK regulates the production of collagenases by synovial fibroblast; and, p38 modulates MMP-3 expression in fibroblasts and osteoclasts [39]. In addition to that, MAPKs may activate the JAK/STAT signaling pathway, which has been described in RA patients, contributing to fibroblast proliferation and osteoclastogenesis [57]. Our data agree with previous reports [45,55,57] where phosphorylation of MAPKs and STAT-3 were increased in CIA mouse group and, positively related to the severity of synovitis, whereas dietary OLE supplementation reduced significantly both MAPKs and STAT-3 activation at transcriptional level. Collectively, our data suggest that dietary OLE may repress cytokines production interfering negatively with pJNK, pp38, pERK MAPKs and pSTAT-3 signaling pathways.

Nrf-2 is a key regulator factor in anti-oxidant response, which plays key roles in cellular defense by modulating HO-1 transcription, enhances ROS removal and mitigates Th-17 cell-mediated inflammation [53]. Besides*,* it has been documented that in knockout mouse Nrf-2 deficiency accelerates the degeneration and joint damage in arthritis disease [58]. In the present study, accordingly with previous reports [45,55,57,58,59], expression of both, Nrf-2 and HO-1, was decreased in the arthritic group, by contrast, dietary OLE could restore Nrf-2 and HO-1 expressions, conferring a role for Nrf-2/HO-1 axis in the beneficial effects of dietary OLE in this murine model of RA.

## 5. Conclusions

Collectively, our study has demonstrated, for the first time, the antioxidant, anti-inflammatory and immunomodulatory effects of dietary OLE in CIA model, which were accompanied by an important attenuation of RA biomarkers (MMP-3), pro-inflammatory cytokines (IL-6, IL-1β, IL-17, IFN-γ, TNF-α), and inhibition of COX-2, mPGES-1 and iNOS overexpression. The mechanisms underlying these protective effects could be related to the activation of Nrf-2/HO-1 axis and the inhibition of relevant signaling pathways, such as JAK-STAT, MAPKs and NF-κB, controlling the inflammatory mediator’s production.

Overall, our results exhibit preliminary evidence for OLE as a new dietary tool against autoimmune and inflammatory disorders, including RA. Nevertheless, further investigations are needed to provide insights into full biological significance of these results and the influence of secoiridoids and their properties on human autoimmune disorders.

## Data Availability

Not applicable.

## Supplementary Materials

The following are available online at https://www.mdpi.com/article/10.3390/antiox10050650/s1, Figure S1: The purity of the extracted OLE was based on the ^1^H and ^13^C NMR spectra and HPLC analyses.
