# Supplementary material for: Dietary Oleocanthal Supplementation Prevents Inflammation and Oxidative Stress in Collagen-Induced Arthritis in Mice
Source: Antioxidants (Basel). 2021 Apr 23;10(5):650. doi: 10.3390/antiox10050650 (PMC8145376; doi:10.3390/antiox10050650)
Supplement: Supplementary file 1 [file antioxidants-10-00650-s001.zip › antioxidants-1175953-supplementary.pdf]

## Supplementary Materials

A

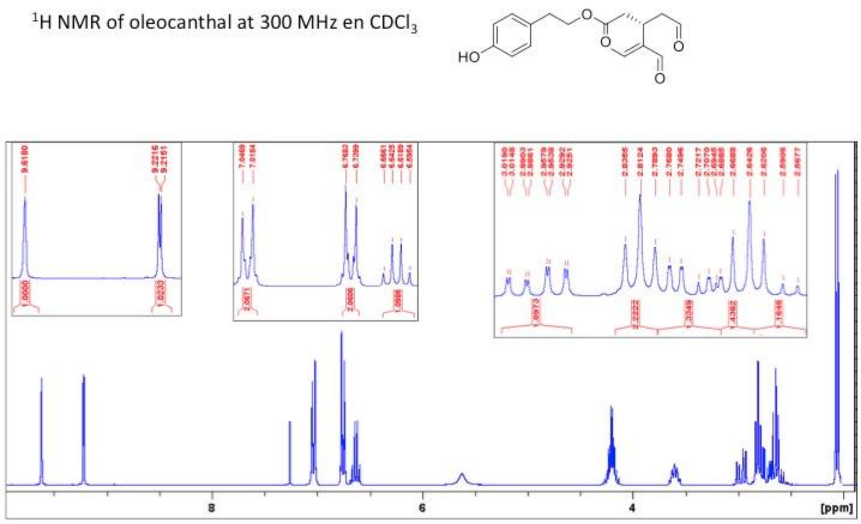


B

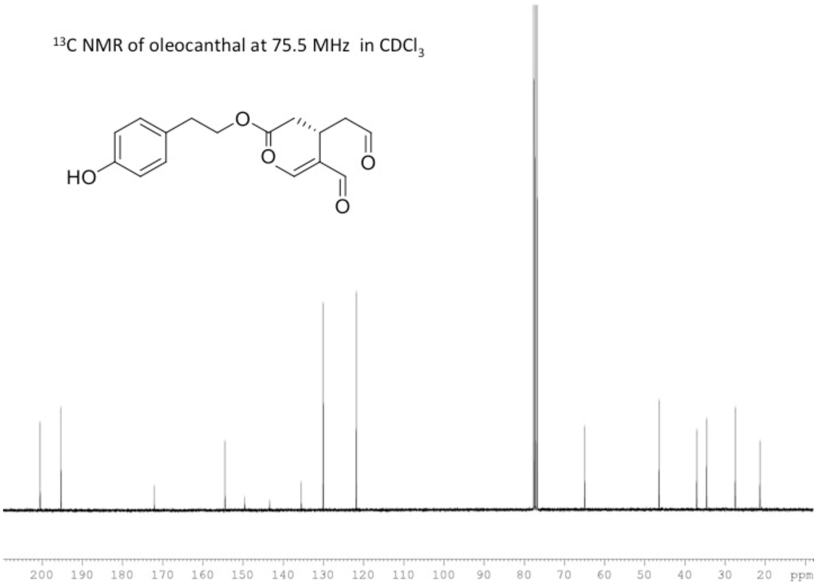


**Figure S1:** The purity of the extracted OLE was based on the $^{1}$H and $^{13}$C NMR spectra and HPLC analyses. (A) $^{1}$H NMR and (B) $^{13}$C NMR of OLE at 300 and 75.5 MHz in CDCl3, respectively. NMR spectra were registered in CDCl3 in a Bruker Avance-300 spectrometer.
